# Excess Weight Among Adults Living in the Coastal Brazilian Amazon: Prevalence, Determinants, and Interventions

**DOI:** 10.1002/ajhb.70231

**Published:** 2026-02-25

**Authors:** Franciane Ferreira Costa, Keyse B. dos Santos Silva, Diego Simeone, João Farias Guerreiro, Rodrigo Alexandre C. Rodrigues, Aldemir B. Oliveira‐Filho

**Affiliations:** ^1^ Programa de Pós‐Graduação em Biologia Ambiental Universidade Federal do Pará Bragança Pará Brazil; ^2^ Grupo de Estudo e Pesquisa em Populações Vulneráveis, Instituto de Estudos Costeiros Universidade Federal do Pará Bragança Pará Brazil; ^3^ Afya Faculdade de Ciências Médicas Bragança Pará Brazil; ^4^ Instituto de Ciências Biológicas, Universidade Federal do Pará Belém Pará Brazil; ^5^ Instituto de Saúde Coletiva, Universidade Federal do Oeste do Pará Santarém Pará Brazil

**Keywords:** Amazon, epidemiology, morbidity, obesity, overweight

## Abstract

Excess weight is a global public health issue related to the accumulation of body fat and can be caused by various factors. This study aimed to determine the prevalence and associated factors of excess weight among adults living in a coastal area of the Brazilian Amazon. Methods. This cross‐sectional study included 407 adults residing in Bragança, Pará, northern Brazil. Socioeconomic, demographic, behavioral, and morbidity data were analyzed using Poisson regression to identify potential associations with excess weight. The prevalence of excess weight was 63.3%. Behavioral factors directly associated with excess weight included avoiding raw salads and boiled eggs. The outcome was associated with factors related to morbidity, such as hypertension and diabetes mellitus. However, walking to work was found to be a protective factor against excessive weight. The high prevalence of excess weight detected in this coastal Amazon region indicates a desire to prioritize this issue in local public health agendas. Implementing individual and community‐based interventions can help reduce health risks and improve the population's quality of life.

**Abbreviations**
BMI: body mass index
NCDs: non‐communicable chronic diseases
PeNSE: National School Health Survey
PNS: National Health Survey
POF: Family Budget Survey
VIGITEL: Health Surveillance System
WHO: World Health Organization

## 1 Introduction

Excess weight is defined as a condition of nutritional status that encompasses both moderate increases in body mass and obesity (Agência Nacional de Saúde Suplementar 2017). Such conditions refer to the accumulation of fat in the body in proportions that harm an individual's health (World Health Organization 2025). With increasingly higher prevalences, excess weight is already considered a global public health problem that mainly affects people living in emerging countries (World Health Organization 2025). So, by the year 2035, around 4 billion adults, teenagers, and children are expected to be excess weight and facing health issues such as obesity, hypertension, and diabetes (WHO Consultation on Obesity 2000; Lobstein et al. 2023). A practical and low‐cost way to obtain a diagnosis of excess weight is by calculating the body mass index (BMI), where weight, given in kilograms, is divided by the square of height, given in meters (kg/m^2^) (WHO Expert Committee on Physical Status 1995). A high BMI (≥ 25) is considered a risk factor for diseases such as type 2 diabetes, hypertension, stroke, and various types of cancer. These morbidities are part of the group of non‐communicable chronic diseases (NCDs), more severe diseases responsible for 54.7% of the deaths recorded in Brazil in 2019 (Martins et al. 2019).

The nutritional status of the Brazilian population has been monitored through nationally representative surveys conducted periodically by the federal government, such as the Family Budget Survey (POF), the National Health Survey (PNS), and the National School Health Survey (PeNSE). According to these surveys, the prevalence of malnutrition in children and adults has seen a rapid decline in recent decades. On the other hand, overweight and obesity have increased in the Brazilian population, especially among adults. The Health Surveillance System (VIGITEL) is another widely used national monitoring tool. In 2006, its first year of data collection, VIGITEL reported an obesity prevalence of 11.8% among adults (≥ 18 years) living in the 27 Brazilian capitals (Ministério da Saúde. Secretaria de Vigilância em Saúde e Ambiente 2024). By 2023, nearly two decades later, the prevalence of obesity had more than doubled (24.3%), and over half of adults were classified as overweight (61.4%) (Ministério da Saúde 2023).

The excessive accumulation of body fat is a multifactorial health problem with a quite complex etiology that is, it can result from the interaction of physiological, genetic, behavioral, and psychological factors. However, factors related to social determinants exert a significant influence on this weight gain process (Associação Brasileira para o Estudo da Obesidade e da Síndrome Metabólica (ABESO) 2016; Branca and Nikogosian 2007; Oliveita et al. 2003). In the northeast of Brazil, two distinct studies showed associations between excess weight and age group, economic class, and marital union. Through the data collected in PNS, another study detected low education and race/color as risk factors for obesity (Melo et al. 2020; Correia et al. 2011; Ferreira et al. 2019). In the Amazon (northern Brazil), most epidemiological studies related to excess weight have been conducted in capitals, such as Rio Branco and Belém (Lino et al. 2011; De Araújo Pinto et al. 2018). In the Brazilian state of Pará, studies directed at the occurrence and factors associated with excess weight have been developed with people living in the metropolitan region of Belém (urban area), overlooking the nutritional status of people living in small and medium‐sized municipalities, as well as factors that contribute to weight gain in these populations living in areas distant from the metropolitan region (Ferreira et al. 2019; Borges et al. 2008).

Considering the different lifestyles of people residing in various areas of the Brazilian Amazon, especially in the state of Pará, the present study detected the prevalence and factors associated with excess weight in a sample of the adult population residing in the coastal municipality of Bragança, Pará, northern Brazil.

## 2 Materials and Methods

### 2.1 Study Design and Participants

**FIGURE 1:**
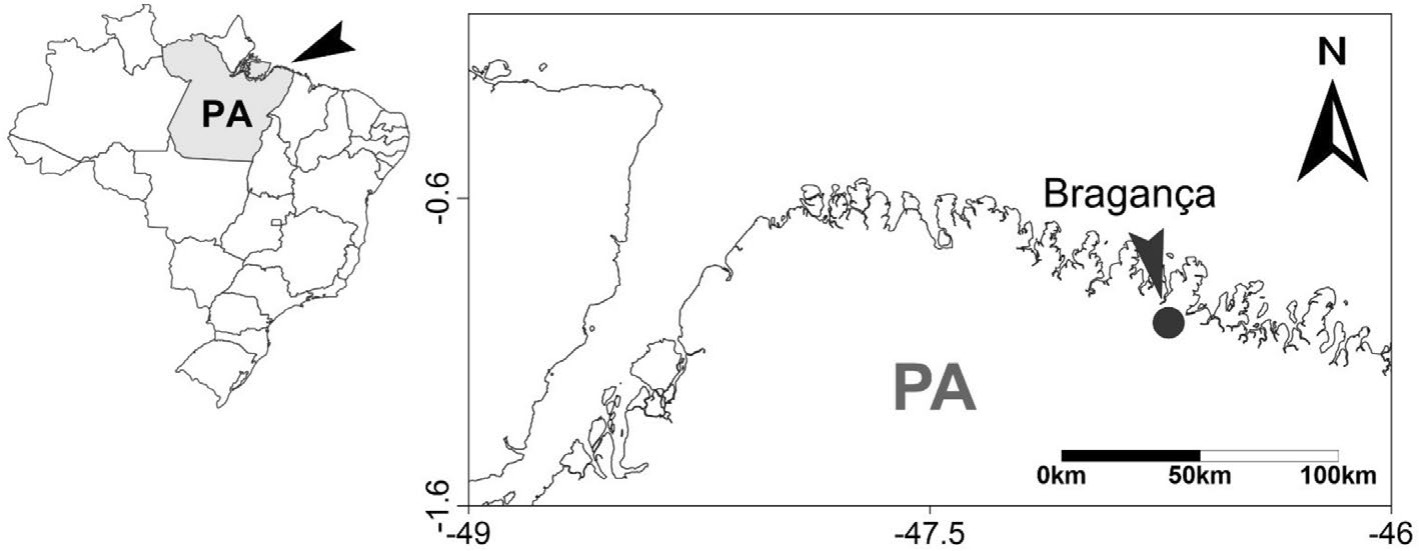
Geographical location of the municipality of Bragança, Brazilian state of Pará (PA), Amazon region.

This cross‐sectional study used data obtained from the project “Cardiovascular Risk in a Municipality of the Northern Coast of Brazil,” developed by the Institute of Health Sciences of the Federal University of Pará and the Municipal Government of Bragança. The collection of socioeconomic and behavioral data was based on a household survey and took place from March to August 2018, in the urban and rural areas of Bragança, a municipality located in the state of Pará (PA), 210 km from the capital Belém, on the coast of the Brazilian Amazon (Figure 1).

### 2.2 Sample Size

According to the Demographic Census of the Brazilian Institute of Geography and Statistics (2010), the population of Bragança in the target age group of the study (30–60 years) was 33 397 inhabitants, with 20 710 in the urban area (62%) and 12 687 in the rural area (38%) (IBGE—Instituto Brasileiro de Geografia e Estatística 2024). Considering a confidence level of 95% and a design error of 5%, a sample size of 395 participants was calculated, with 245 in the urban area and 150 in the rural area. A total of 407 adults constituted the final sample. The number of observations evaluated here constitutes a subset of the sample, representing the universe of people over 30 years old, calculated to portray the entirety of adults in the municipality of Bragança.

### 2.3 Sampling

The sampling was done randomly, stratified by census sector, gender, and age group. This research excluded individuals who had any physical or mental impairment that would prevent them from undergoing any of the prescribed data collection procedures, as well as those who refused to sign the Free and Informed Consent Form.

### 2.4 Outcome

The analysis of the participants' nutritional status was conducted through the measurement of anthropometric indices (weight and height) used to calculate the BMI. Weight and height measurements were taken only once. According to the World Health Organization (WHO), adults can be classified as: underweight (< 18.5 kg/m^2^), normal weight (≥ 18.5 to < 25 kg/m^2^), overweight (≥ 25 to < 30), and obesity (≥ 30 kg/m^2^) (17). In this study, overweight/obesity were represented by the variable excess weight, considering adults with a BMI ≥ 25 kg/m^2^ (WHO Consultation on Obesity 2000). Excess weight was the main outcome of the study. To obtain the body weight, a Balmak brand scale, model Slimbasic 200, portable, with a maximum capacity of 200 kg and a minimum variation of 0.1 kg, was used, placed on a flat surface in the participant's home. During weighing, the individual remained in an upright position, arms extended along the body, without shoes, and wearing light clothing. For measuring height, a SECA stadiometer, model 213, with a length of 205 cm and 1 mm intervals, was used, with the participant in an upright position, without shoes, feet together, arms extended along the body, looking straight ahead. The stadiometer, fixed on a smooth surface, on a wall without a baseboard or portal, was positioned firmly against the top of the head, slightly compressing the hair.

### 2.5 Variables

The independent variables were displayed categorically, being classified into:

Socioeconomic and demographic variables. Sex (male and female), age group (30–39, 40–49, 50–59, and 60 or more), marital status (married + stable union and single + divorced + widowed), education (illiterate, completed elementary school, completed high school, and higher education), race/ethnicity (indigenous, yellow, brown, white, and black), income per minimum wage—using the Brazil 2018 Criterion—(up to 1—including irregular income, more than 1 up to 2, more than 2 up to 3, more than 3 up to 4, and more than 4), housing and basic sanitation—wall (brick/masonry and others), water supply (general network and others), waste disposal (public collection and others), domestic sewage disposal (general network and others).

Behavioral variables. Weekly food consumption—white rice, beans, pasta, cassava flour, raw salad, cooked salad, fried egg, boiled egg, tubers, fruits, bread, whole milk, fresh beef, chicken, fresh fish, salted fish, shellfish, açaí, soda, artificial juice, cakes and pies, fried and baked snacks or processed meats (never, 1–2 times, 3–4 times, and 5–7 times), industrialized sweetener or seasoning (yes and no), walking to work, walking for leisure, cycling to work, cycling for leisure, or practicing sports (yes and no), currently consumes alcoholic beverages, or currently consumes tobacco (yes and no). The dietary pattern was assessed according to the Food Frequency Questionnaire, based on a survey that uses the ELSA‐Brasil study database, employing Multiple Correspondence Analysis and Cluster Analysis, identifying 4 dietary patterns: “traditional”; “fruits and vegetables”; “bakery”; and “diet/light” (Cardoso et al. 2016).

Physical activity. The determination of the level of physical activity was based on the WHO's global guidelines, which classify adults aged 18 to 64 as “active” when they engage in at least 150 min of moderate physical activity per week, or 75 min or more of vigorous physical activity. If they practice less, they are considered “somewhat active.” And those who do not engage in any type of physical activity are considered “inactive” (World Health Organization 2010).

Smoking. A participant is considered a “smoker” if they reported having smoked at least 100 cigarettes in their lifetime and still smoke; an “ex‐smoker” if they smoked 100 cigarettes in their lifetime and do not smoke anymore; and a “non‐smoker” if they have never smoked or have smoked fewer than 100 cigarettes in their lifetime. To estimate alcohol consumption, the National Center for Health Statistics questionnaire was used, classifying the participant as: “never used alcohol,” “former user,” “moderate user,” and “excessive user” (Santana 2017).

Morbidity. Diabetes mellitus and hypertension were the morbidities studied. Diabetes mellitus was diagnosed using a biochemical examination or when the individual reported having diabetes and utilizing hypoglycemic medication. A 5 mL sample of the participant's peripheral venous blood was obtained after a 12‐h fast and used to evaluate fasting blood glucose and glycated hemoglobin. Whereas a case was defined when the participant's blood glucose was greater than or equal to 126 mg/dL, and glycated hemoglobin levels were at or above 6.5%. Blood pressure was measured according to the sixth Ambulatory Blood Pressure Monitoring Guidelines of the Brazilian Society of Cardiology, with abnormal values considered as: 24‐h average ≥ 130/80 mmHg; daytime average ≥ 135/85 mmHg; nighttime average ≥ 120/70 mmHg. Furthermore, the nocturnal decline can be classified as: absent or an increase in blood pressure, when less than 0%; attenuated, between 0% and 10%; normal, between 10% and 20%; and pronounced, when above 20%. They were considered abnormal, and the information from those who claimed to use antihypertensive medication was considered (Brandão et al. 2018). The G‐Tech LA800 Digital Arm Blood Pressure Monitor (model LA800) was used to take blood pressure measurements.

### 2.6 Data Analysis

Descriptive analyses were performed to characterize and summarize the distribution of the study variables. Subsequently, bivariate analyses using simple Poisson regression were carried out to evaluate potential associations between excess weight and the independent variables. In the adjusted model (multivariate analysis), variables with a *p* value < 0.20 in the crude analysis were considered for inclusion. The final model was determined following a conceptual hierarchical approach, prioritizing variables of theoretical relevance while retaining those that remained statistically significant (*p* < 0.05). Overdispersion was evaluated by comparing the residual deviance with the model's degrees of freedom; as the ratio was approximately 1, no substantial overdispersion was detected, supporting the use of the Poisson model. Multicollinearity among independent variables was assessed using the Variance Inflation Factor (VIF), and all variables presented VIF values below 5, indicating no significant collinearity issues. Multivariate Poisson regression with robust standard error adjustment was then applied, and results were expressed as prevalence ratios (PR), *p* value and 95% confidence intervals (CI). Data analyses were performed using R software, version 4.3.1.

### 2.7 Ethical Aspects

This study was submitted to the Research Ethics Committee of the Institute of Health Sciences at the Federal University of Pará under the number CAAE94952518.4.0000.0018. All participants signed the Informed Consent Form, and those with excess weight were advised and referred for care in the public health network.

## 3 Results

In total, 450 participants were approached. However, 43 participants were excluded due to gaps in filling out the questionnaire, refusal to complete it in full, and withdrawal from participation. The sample consisted of 407 participants, with the majority being male (57.1%), brown (83.3%), in the age range of 30–39 years (30.7%), in a marital relationship (76.2%), having completed high school (35.6%), and with a monthly income of up to a minimum wage (58.9%). Table 1 presents the prevalences of different nutritional states detected in the population sample, including the prevalence of participants with excess weight (63.3%). The prevalence of individuals with excess weight was much higher than the rate of eutrophic individuals, and the prevalence of obese individuals was almost 10 times higher than that of underweight individuals.

**TABLE 1:** Nutritional status of the sample of adults in the municipality of Bragança who participated in this study.

Information	*N*	%	95% CI	*p* **
Nutritional states				
Low weight	8	2.0	1.0–3.0	
Adequate weight (eutrophic)	141	34.7	33.5–36.1	
Overweight	184	45.2	43.6–47.6	
Obesity	74	18.1	15.5–21.5	0.01
Obesity I	54	13.2	11.1–16.1	
Obesity II	13	3.1	0.4–6.7	
Obesity III	7	1.8	0.0–5.3	
Excess weight				
Yes	258	63.3	61.7–66.0	—
No	149	36.7		

Abbreviation: 95% CI = 95% confidence interval.

** *p* value generated from the multivariate regression model.

The variables related to socioeconomic and demographic issues were not associated with excess weight. The behavioral variables of never consuming raw salad during the week or boiled eggs during the week had a direct association with excess weight, as well as the morbidities of diabetes mellitus and hypertension. However, the behavioral variable of walking to work showed an inverse association with excess weight (*p* < 0.20) (Tables 2 and 3).

**TABLE 2:** Prevalence and crude prevalence ratio (CPR) of adults with excess weight, according to behavioral variables, in the municipality of Bragança, Pará, Brazilian Amazon.

Variables	Sample		Excess weight		CPR	*p* **
	*N*	%	*N*	%	(95% CI)	
Behavioral						
Eating raw salad*						
Never	252	61.9	152	37.4	38.78 (36.35–39.24)	
1–2 times	93	22.9	58	14.3	1.0	0.05
3–4 times	32	7.9	22	5.4	6.05 (4.58–6.89)	
5–7 times	30	7.4	26	6.4	7.37 (5.06–7.56)	
Eat boiled egg*						
Never	293	71.9	198	48.7	50.04 (47.61–50.33)	
1–2 times	87	21.4	44	10.8	1.0	0.01
3–4 times	18	4.4	9	2.2	3.19 (1.56–3.73)	
5–7 times	9	2.2	7	1.7	3.24 (0.75–4.19)	
Walk to work*						
No	171	42.0	118	28.9	1.0	0.04
Yes	236	57.9	140	34.4	35.25 (33.66–35.39)	

* Weekly food consumption.

** Poisson regression: bivariate analysis.

**TABLE 3:** Prevalence and crude prevalence ratio (CPR) of adults with excess weight, according to morbidities, in the municipality of Bragança, Pará, Brazilian Amazon.

Variables	Sample		Excess weight		CPR	*p* **
	*N*	%	*N*	%	(95% CI)	
Morbidity						
Diabetes mellitus						
No	287	95.1	239	58.7	1.0	0.005
Yes	20	4.9	19	4.7	6.23 (3.54–6.74)	
Hypertension						
No	379	93.1	234	57.5	1.0	0.02
Yes	28	6.9	24	5.9	7.28 (4.82–7.69)	

** Poisson regression: bivariate analysis.

The adjusted prevalences and PR demonstrated that the weekly frequency of raw salad and boiled egg consumption (never) was associated with elevated BMI, compared to the reference category (5–7 times/week) (Table 4). The lower the weekly frequency of raw salad and boiled egg consumption, the higher the frequency of adults with excess weight. The morbidities diabetes mellitus and hypertension were also associated with the outcome (Table 4). People who had these comorbidities showed a higher frequency of excess weight compared to those who did not have such comorbidities. Such variables remained associated with the outcome *p* < 0.05. The variables not associated with excess weight were made available in Table S1.

**TABLE 4:** Factors associated with excess weight among adults in the municipality of Bragança, Pará, Brazilian Amazon.

Variables	Adjusted PR	95% CI	*p* **
Eat raw salad*			
Never	1.34	1.01–1.79	0.04
1–2 times	1		
3–4 times	1.18	0.87–1.60	0.27
5–7 times	0.97	0.81–1.17	0.79
Eat boiled egg*			
Never	1.36	1.12–1.67	0.002
1–2 times	1		
3–4 times	0.91	0.59–1.42	0.79
5–7 times	1.44	0.87–2.37	0.14
Walk to work			
No	1		
Yes	0.87	0.75–1.01	0.08
Diabetes mellitus			
No	1		
Yes	1.33	0.96–1.83	0.07
Hypertension			
No	1		
Yes	1.23	0.92–1.64	0.14

* Weekly food consumption.

** Poisson regression: multivariate analysis.

## 4 Discussion

This study is one of the first epidemiological reports on excess weight in a sample of adults residing in the interior of the Amazon. The findings demonstrated that the prevalence of excess weight was much higher compared to the capital of the state of Pará—Belém (57.7%)—and other Brazilian capitals (55.7%) in 2018 (Ministério da Saúde 2019). In this same survey, the capitals of northern Brazil showed similar obesity prevalence, reinforcing that the region is following the global process of nutritional transition, that is, the prevalence of malnutrition is decreasing, while the prevalence of obesity is increasing at an accelerated pace (Ministério da Saúde 2019; Baptista and Da Cruz 2004).

Throughout life, diet and nutrition are important aspects in promoting and maintaining good health (WHO 2003). Natural foods, which are those obtained directly from plants or animals—such as leaves, fruits, eggs, and milk—without undergoing any changes after leaving nature, generally have low calorie content and are sources of fiber and various nutrients; when combined with small quantities of other animal‐origin foods, they form the basis of a nutritionally balanced diet (Ministério da Saúde 2014). The recommendation is that a person consume at least five servings of fruits and vegetables daily (WHO 2003).

The Amazon region is known for the abundance of its natural resources, enabling a diet traditionally based on foods from the rivers, the forest, and sustainable extraction, such as fish, cassava, Brazil nuts, açaí, cupuaçu, buriti, and countless other native fruits (Gama et al. 2022). However, most participants (61.9%) in this study do not consume raw salad vegetables. This finding corroborates the results of other studies conducted in Amazon municipalities that identified a high consumption of ultra‐processed foods, in addition to excessive consumption of fried foods (Nogueira et al. 2021; Da Cunha et al. 2022). A study conducted in the capital of the state of Pará found that the factors hindering adequate consumption of both fruits and vegetables are perishability and lack of habit, and the higher the declared income of individuals, the greater the chances of regularly consuming fruits and vegetables (Ferreira 2021). Unlike other areas of the Amazon, where barriers to consuming fruits, vegetables, and greens are linked to periods of river flooding and drought (Gama et al. 2022; Da Silva et al. 2020), the situation in this study differs. The low intake of these foods is directly associated with excess weight and represents a warning, as the state of Pará has recorded a low frequency of fruit and vegetable consumption in national surveys (Ministério da Saúde 2023, 2020).

Another dietary variable that showed an association with the outcome was the non‐consumption of boiled eggs, which suggests an increasingly frequent modification in main meals from foods considered healthy to processed and ultra‐processed foods. Investigations conducted in traditional Amazonian communities have already described the low level of egg consumption. The deficit in protein intake was primarily caused by replacing eggs with other protein sources, such as jerky, and by the inclusion of foods that are considered uncommon in the investigated communities (region of the middle Solimões River) in the Amazon region (Gama et al. 2022; Mendes et al. 2012). The egg is considered a source of high‐quality and low‐cost protein (Puglisi and Fernandez 2022). When consumed at breakfast, it helps reduce appetite for subsequent meals, consequently aiding in weight reduction and maintenance (Vander Wal et al. 2005). And when associated with a plant‐based diet, it presents a double benefit for people with metabolic syndrome. It can elevate HDL cholesterol levels, thereby aiding in the prevention of cardiovascular diseases. Another benefit is the high concentrations of essential plasma compounds such as lutein, zeaxanthin, and choline, found in low quantities in this population, which can help protect against chronic diseases such as type 2 diabetes, cardiovascular diseases, Alzheimer's, and so on (Thomas et al. 2022).

Walking, a low‐cost exercise with various health benefits, is accessible to everyone (Melo 2012). In this study, the behavioral variable of walking to work was found to be inversely associated with the outcome, reinforcing the VIGITEL data on the frequency of physical activity in the capitals of Brazilian states and the Federal District, where Belém stands out as the Amazonian capital with the best average percentage of adults who engage in physical activity during their commute to work or school from 2006 to 2023 (Ministério da Saúde 2024). The establishment of a routine of walking to work in the studied population can be supported by two main factors: the Amazonian climate, which is predominantly humid tropical or subhumid (according to the Köppen classification adaptation) and features two well‐defined seasons (dry and rainy) that allow for walking to work for a significant part of the year, and the geomorphology of the municipality, which lies within the geomorphological domain of the coastal plain of northeastern Pará (Souza Filho and El‐Robrini 1996). Flat environments facilitate walking and other physical activities. Such behaviors related to daily commuting to work reduce the risk of cardiovascular diseases, particularly stroke (Hu et al. 2005; Hamer and Chida 2008).

Furthermore, diabetes mellitus and hypertension are NCDs of high relevance to global public health. In this study, excess weight was associated with these morbidities, reaffirming that this combination is generally a reality for excess‐weight individuals. These conditions contribute to the development of various disorders, collectively referred to as metabolic syndrome (Ferreira et al. 2019; Sousa et al. 2021; Silva et al. 2006). These morbidities are also increasing in other Amazonian populations, such as the indigenous peoples of the Munduruku, Tembé, and Kaiapó tribes (De Souza Benedito et al. 2025). A health education intervention for users with hypertension and diabetes at a Basic Health Unit in the municipality of Redenção (state of Pará) showed improved self‐management and self‐care. It was feasible to authenticate features of these morbidities, as well as their effective management, hence reducing the probability of consequences from these disorders (dos Santos et al. 2023).

In 2011, the Ministry of Health of Brazil launched the Strategic Action Plan for the Fight Against NCDs (2011–2022) with the aim of developing and implementing integrated, effective, sustainable, and evidence‐based public policies for the prevention and control of NCDs. In these actions, excess weight was treated as a modifiable risk factor for NCDs, along with smoking, excessive alcohol consumption, physical inactivity, and diet (Ministério da Saúde 2021). In the Brazilian Amazon, one of the main alternatives to combat obesity is the “Zero Obesity” program, launched in 2020 by the government of the state of Pará, where users undergo gastroplasty, that is, the reduction of stomach size, which, combined with other measures, leads to the weight loss that the patient needs (Abreu 2020). In the international Amazon region, few countries have developed comprehensive policies and strategies for preventing obesity. Peru has a national plan, like Brazil's, aimed at addressing NCDs to prevent excess weight. In Ecuador, the regulation of a front warning system on labels, indicating high levels of sodium, sugar, and/or fat, allows consumers to better classify products according to their nutritional quality. This measure was widely understood and recognized as an influence on food purchasing decisions (Palacios et al. 2021).

Studies conducted in other Brazilian regions indicate socioeconomic and demographic factors, such as income, age group, and color, are associated with the process of excessive weight gain. However, the present study did not detect associations of this nature (Melo et al. 2020; Correia et al. 2011; Ferreira et al. 2019; Sousa et al. 2021). Likely, the Amazon region still maintains specific regional sociocultural aspects, such as some traditional dietary habits and lifestyles capable of modulating the impact of these variables on excess weight, making it different from other regions of Brazil, where the process of urbanization and nutritional transition has a greater influence. Local dietary practices, shaped by indigenous peoples and immigrants, apparently maintain a characteristic pattern that can reduce these associations (Araújo et al. 2010; Coimbra et al. 2021). In this study, the association between excess weight and educational level was detected, but some research has already recorded this direct relationship in men (Barbosa et al. 2009; Gigante et al. 2009; Júnior and Verona 2019). For women, this link appears to be inversely proportionate. Thus, a higher level of knowledge lowers the likelihood of being excess weight (Júnior and Verona 2019). Women who have studied for less than 5 years are more likely to be excess weight than those with greater education (Correia et al. 2011; Linhares et al. 2012).

This study has limitations that must be considered. Since it is a cross‐sectional design, the analysis of the relationships between the variables and the outcome becomes limited, making it impossible to infer causal relationships. The cutoff values established for calculating BMI for the diagnosis of excess weight also prove to be a limiting factor, as there is evidence that ethnic aspects can influence these anthropometric measures. However, the parameters established by the WHO are not specific to these groups (Sellen 1998; Simões et al. 2013). Even so, the findings of this study can assist in the planning and implementation of measures to control and prevent excess weight and related comorbidities.

## 5 Conclusions

This study identified the prevalence of excess weight and the factors contributing to it among adult residents in a municipality in the coastal region of the Amazon. Being excess weight was linked to a diet lacking vegetable salads and eggs. The finding means that public health programs that focus on nutrition should be initiated, but they should also consider the region's cultural and social norms. Existing initiatives, such as the Food Guide for the Brazilian Population, should be used more frequently and adapted, if necessary, to educate the population about healthy eating and proper nutrition. NCDs, such as diabetes and hypertension, have also been associated with excess weight, so it's important to strengthen public policies created by the federal government, such as Primary Health Care and the Family Health Strategy. The practice of walking to work was identified as a protective factor against excess weight, motivating public policies aimed at improving the physical and structural aspects of the city and raising awareness through informational campaigns about the importance of adopting a more active lifestyle for these individuals. This information offers possibilities for individual and collective interventions for a healthier and more balanced diet, considering the practice of daily physical activities, which will enable a lower risk of health issues and a better quality of life for the population of this Brazilian region and others with similar sociocultural and economic characteristics.

## Author Contributions

All authors contributed to the development of study. Conceptualization: R.A.C.R., J.F.G., and A.B.O‐.F. Data curation: F.F.C., K.B.d.S.S., and A.B.O‐.F. Formal analysis: D.S. and A.B.O‐.F. Methodology: R.A.C.R. and J.F.G. Project administration: R.A.C.R. Writing – original draft: F.F.C. Writing – review and editing: K.B.d.S.S., D.S., R.A.C.R., J.F.G., and A.B.O‐.F. All authors read and approved the final manuscript.

## Funding

The authors have nothing to report.

## Ethics Statement

This study was part of the project “Cardiovascular Risk in a Municipality of the North Coast of Brazil,” promoted by the Postgraduate Program in Health, Environment and Society of the Amazon, from the Institute of Health Sciences of the Federal University of Pará, approved by the Committee Research Ethics with the number 94952518.4.0000.0018, in accordance with Resolution 466/2012 of the National Health Council/Ministry of Health of Brazil, which considers several international documents, including the Declaration of Helsinki.

## Consent

All participants signed an informed consent after a detailed explanation of the study objectives and procedures.

## Conflicts of Interest

The authors declare no conflicts of interest.

## Supporting information


**Table S1:** Prevalence and crude prevalence ratio (CPR) of excess weight in adults residing in the Brazilian municipality of Bragança, according to socioeconomic and demographic variables, housing and basic sanitation, dietary habits and behavioral patterns.

## Data Availability

The data that support the findings of this study are available on request from the corresponding author. The data are not publicly available due to privacy or ethical restrictions.

## References

[ajhb70231-bib-0001] Abreu, G. 2020. “Governo do Estado realiza primeira cirurgia bariátrica do Programa Obesidade Zero.” In: Agencia Pará. Belém, Pará, 01 out. https://agenciapara.com.br/noticia/22511/governo‐do‐estado‐realiza‐primeira‐cirurgia‐bariatrica‐do‐programa‐obesidade‐zero#:~:text=O%20paciente%20precisa%20perder%20peso,m%C3%A9dico%20cirurgi%C3%A3o%20Carlos%20Armando%20Ribeiro.&text=O%20primeiro%20passo%20para%20o,que%20dever%C3%A1%20comparecer%20%C3%A0%20consulta.&text=S%C3%A3o%20candidatas%20%C3%A0%20cirurgia%20bari%C3%A1t.

[ajhb70231-bib-0002] Agência Nacional de Saúde Suplementar . 2017. Manual de diretrizes para o enfrentamento da obesidade na saúde suplementar brasileira. Agência Nacional de Saúde Suplementar. https://www.gov.br/ans/pt‐br/assuntos/noticias/beneficiario/ans‐lanca‐o‐manual‐de‐diretrizes‐para‐o‐enfrentamento‐da‐obesidade‐na‐saude‐suplementar‐brasileira.

[ajhb70231-bib-0003] Araújo, M. S. , T. H. Costa , B. A. Schmitz , L. M. Machado , and W. R. Santos . 2010. “Fatores associados ao sobrepeso e à adiposidade central em trabalhadores urbanos cobertos pelo Programa Alimentar para Trabalhadores da Amazônia Brasileira.” Revista Brasileira de Epidemiologia 13: 425–433. 20857029 10.1590/s1415-790x2010000300006

[ajhb70231-bib-0004] Associação Brasileira para o Estudo da Obesidade e da Síndrome Metabólica (ABESO) . 2016. Diretrizes Brasileiras de Obesidade 2016. Associação Brasileira para o Estudo da Obesidade e da Síndrome Metabólica. https://abeso.org.br/diretrizes/.

[ajhb70231-bib-0005] Baptista, T. J. R. , and A. M. Da Cruz . 2004. “Obesidade: saúde, doença e efeitos do treinamento.” Pensar a Prática 7, no. 1: 103–120.

[ajhb70231-bib-0006] Barbosa, J. M. , P. C. Cabral , P. I. C. de Lira , and T. M. de Menezes Toledo Florêncio . 2009. “Fatores socioeconômicos associados ao excesso de peso em população de baixa renda do Nordeste brasileiro.” Archivos Latinoamericanos de Nutrición 59, no. 1: 22–29. 19480340

[ajhb70231-bib-0007] Borges, H. P. , N. d. C. Cruz , and E. C. Moura . 2008. “Associação entre hipertensão arterial e excesso de peso em adultos, Belém, Pará, 2005.” Arquivos Brasileiros de Cardiologia 91: 110–118. 10.1590/s0066-782x2008001400007 18709261

[ajhb70231-bib-0008] Branca, F. , and H. Nikogosian . 2007. The Challenge of Obesity in the WHO European Region and the Strategies for Response: Summary, edited by T. Lobstein . World Health Organization.

[ajhb70231-bib-0009] Brandão, A. A. , A. M. Feitosa , C. E. P. de Figueired , et al. 2018. “6ª Diretrizes de monitorização ambulatorial da pressão arterial e 4ª Diretrizes de monitorização residencial da pressão arterial.” Arquivos Brasileiros de Cardiologia 110, no. 5 supl 1: 1–29.29538519

[ajhb70231-bib-0010] Cardoso, L. d. O. , M. S. Carvalho , O. G. Cruz , et al. 2016. “Eating Patterns in the Brazilian Longitudinal Study of Adult Health (ELSA‐Brasil): An Exploratory Analysis.” Cadernos de Saúde Pública 32: e00066215. 27192025 10.1590/0102-311X00066215

[ajhb70231-bib-0011] Coimbra, C. E. , F. G. Tavares , A. A. Ferreira , et al. 2021. “Socioeconomic Determinants of Excess Weight and Obesity Among Indigenous Women: Findings From the First National Survey of Indigenous People's Health and Nutrition in Brazil.” Public Health Nutrition 24, no. 7: 1941–1951. 32476634 10.1017/S1368980020000610 PMC8094432

[ajhb70231-bib-0012] Correia, L. L. , D. M. I. da Silveira , A. C. e. Silva , et al. 2011. “Prevalência e determinantes de obesidade e sobrepeso em mulheres em idade reprodutiva residentes na região semiárida do Brasil.” Ciência & Saúde Coletiva 16: 133–145. 21180822 10.1590/s1413-81232011000100017

[ajhb70231-bib-0013] Da Cunha, S. S. , C. De Araújo Amaral , and G. T. R. Monteiro . 2022. “Padrões alimentares e doenças crônicas em inquérito com adultos na Amazônia.”

[ajhb70231-bib-0014] Da Silva, L. S. , H. da Silva Alves , D. W. A. G. N. E. R. Silva , and M. L. P. C. Romano . 2020. “Alimentação na várzea amazônica: estudo dos hábitos alimentares de famílias ribeirinhas do município de Alenquer‐PA.” Revista Ciências da Sociedade 4, no. 7: 177–206.

[ajhb70231-bib-0015] De Araújo Pinto, A. , A. d. A. Pinto , R. M. d. S. P. Barbosa , M. V. Nahas , and A. Pelegrini . 2018. “Prevalência de excesso de peso e fatores demográficos e econômicos associados em adolescentes de Manaus, a maior cidade do norte do Brasil.” Revista de Atenção à Saúde 16, no. 55: 64–71.

[ajhb70231-bib-0016] De Souza Benedito, J. C. , A. L. Martins , E. F. Teston , and E. Girotto . 2025. “Doenças Crônicas Não Transmissíveis Na População Indígena Brasileira: Uma Revisão Integrativa.” SciELO Preprints: 1–42. 10.1590/SciELOPreprints.12019.

[ajhb70231-bib-0017] dos Santos, S. C. , K. F. S. Delmondes , I. A. Silva , et al. 2023. “A PRÁTICA DE EDUCAÇÃO EM SAÚDE A PORTADORES DE HIPERTENSÃO E DIABETES NA ATENÇÃO PRIMÁRIA.” Revista Ibero‐Americana de Humanidades, Ciências e Educação 9, no. 5: 971–980. 10.51891/rease.v9i5.9839.

[ajhb70231-bib-0018] Ferreira, A. P. d. S. , C. L. Szwarcwald , and G. N. Damacena . 2019. “Prevalência e fatores associados da obesidade na população brasileira: estudo com dados aferidos da Pesquisa Nacional de Saúde, 2013.” Revista Brasileira de Epidemiologia 22: e190024. 30942330 10.1590/1980-549720190024

[ajhb70231-bib-0019] Ferreira, V. d. M. 2021. “Avaliação de fatores determinantes do consumo de frutas e hortaliças por acadêmicos da área da saúde de uma universidade pública em Belém, Pará.”

[ajhb70231-bib-0020] Gama, A. S. M. , L. P. Corona , B. M. Tavares , and S. R. Secoli . 2022. “Padrões de consumo alimentar nas comunidades ribeirinhas da região do médio rio Solimões‐Amazonas‐Brasil.” Ciência & Saúde Coletiva 27: 2609–2620. 35730832 10.1590/1413-81232022277.20362021

[ajhb70231-bib-0021] Gigante, D. P. , E. C. de Moura , and L. M. V. Sardinha . 2009. “Prevalência de excesso de peso e obesidade e fatores associados, Brasil, 2006.” Revista de Saúde Pública 43: 83–89. 19936502 10.1590/s0034-89102009000900011

[ajhb70231-bib-0022] Hamer, M. , and Y. Chida . 2008. “Active Commuting and Cardiovascular Risk: A Meta‐Analytic Review.” Preventive Medicine 46, no. 1: 9–13. 17475317 10.1016/j.ypmed.2007.03.006

[ajhb70231-bib-0023] Hu, G. , C. Sarti , P. Jousilahti , K. Silventoinen , N. C. Barengo , and J. Tuomilehto . 2005. “Leisure Time, Occupational, and Commuting Physical Activity and the Risk of Stroke.” Stroke 36: 1994–1999. 16081862 10.1161/01.STR.0000177868.89946.0c

[ajhb70231-bib-0024] IBGE—Instituto Brasileiro de Geografia e Estatística . 2024. “Panorama: Bragança, Pará.” https://cidades.ibge.gov.br/brasil/pa/braganca/panorama.

[ajhb70231-bib-0025] Júnior, C. S. D. , and A. P. Verona . 2019. “Excesso de peso, obesidade e educação no Brasil.” Saude (Santa Maria) 45: 1–8.

[ajhb70231-bib-0026] Linhares, R. d. S. , B. L. Horta , D. P. Gigante , J. S. Dias‐da‐Costa , and M. T. A. Olinto . 2012. “Distribuição de obesidade geral e abdominal em adultos de uma cidade no Sul do Brasil.” Cadernos De Saude Publica 28: 438–447. 22415176 10.1590/s0102-311x2012000300004

[ajhb70231-bib-0027] Lino, M. Z. R. , P. T. Muniz , and K. S. Siqueira . 2011. “Prevalência e fatores associados ao excesso de peso em adultos: inquérito populacional em Rio Branco, Acre, Brasil, 2007‐2008.” Cadernos de Saúde Pública 27: 797–810. 21603763 10.1590/s0102-311x2011000400019

[ajhb70231-bib-0028] Lobstein, T. , R. Jackson‐Leach , J. Powis , H. Brinsden , and M. Gray . 2023. World Obesity: Atlas 2023. World Obesity Federation.

[ajhb70231-bib-0029] Martins, S. C. , C. Sacks , W. Hacke , et al. 2019. “Priorities to Reduce the Burden of Stroke in Latin American Countries.” Lancet Neurology 18, no. 7: 674–683. 31029579 10.1016/S1474-4422(19)30068-7

[ajhb70231-bib-0030] Melo, D. G. 2012. “Benefícios da prática da caminhada para os idosos do grupo “terceira idade” de Cavalcante‐GO. 2012.” Trabalho de Conclusão de Curso (Licenciatura em Educação Física). Universidade de Brasília Alto Paraíso‐GO.

[ajhb70231-bib-0031] Melo, S. P. d. S. d. C. , E. Â. P. Cesse , P. I. C. Lira , L. C. C. D. N. Ferreira , A. Rissin , and M. Batista Filho . 2020. “Overweight and Obesity and Associated Factors in Adults in a Poor Urban Area of Northeastern Brazil.” Revista Brasileira de Epidemiologia 23: e200036. 32428190 10.1590/1980-549720200036

[ajhb70231-bib-0032] Mendes, P. M. , T. O. Souza , and A. P. Oliveira . 2012. “Consumo alimentar e disponibilidade de alimentos dos moradores da Ilha de Cotijuba no Bioma Amazônico.” Revista da Universidade Vale do Rio Verde 10, no. 2: 279–288.

[ajhb70231-bib-0033] Ministério da Saúde , ed. 2014. Guia Alimentar para a População Brasileira/Ministério da Saúde, secretaria de atenção à saúde, departamento de atenção Básica. 2nd ed. Ministério da Saúde.

[ajhb70231-bib-0034] Ministério da Saúde . 2019. VIGITEL Brasil 2018: vigilância de fatores de risco e proteção para doenças crônicas por inquérito telefônico: estimativas sobre frequência e distribuição sociodemográfica de fatores de risco e proteção para doenças crônicas nas capitais dos 26 estados brasileiros e no Distrito Federal em 2018. Ministério da Saúde.

[ajhb70231-bib-0035] Ministério da Saúde . 2020. Vigitel Brasil 2019: Vigilância de fatores de risco e proteção para doenças crônicas por inquérito telefônico: estimativas sobre frequência e distribuição sociodemográfica de fatores de risco e proteção para doenças crônicas nas capitais dos 26 estados brasileiros e no Distrito Federal em 2019. Ministério da Saúde.

[ajhb70231-bib-0036] Ministério da Saúde . 2021. VIGITEL Brasil 2020: vigilância de fatores de risco e proteção para doenças crônicas por inquérito telefônico: estimativas sobre frequência e distribuição sociodemográfica de fatores de risco e proteção para doenças crônicas nas capitais dos 26 estados brasileiros e no Distrito Federal em 2020. Ministério da Saúde.

[ajhb70231-bib-0037] Ministério da Saúde . 2023. VIGITEL Brasil 2020: vigilância de fatores de risco e proteção para doenças crônicas por inquérito telefônico: estimativas sobre frequência e distribuição sociodemográfica de fatores de risco e proteção para doenças crônicas nas capitais dos 26 estados brasileiros e no Distrito Federal em 2023. Ministério da Saúde.

[ajhb70231-bib-0038] Ministério da Saúde . 2024. “Vigitel Brasil 2006–2023: vigilância de fatores de risco e proteção para doenças crônicas por inquérito telefônico: estimativas sobre frequência e distribuição sociodemográfica de prática de atividade física nas capitais dos 26 estados brasileiros e no Distrito Federal entre 2006 e 2023: prática de atividade física [recurso eletrônico].” Ministério da Saúde, Secretaria de Vigilância em Saúde e Ambiente, Departamento de Análise Epidemiológica e Vigilância de Doenças não Transmissíveis Brasília Ministério da Saúde.

[ajhb70231-bib-0039] Ministério da Saúde. Secretaria de Vigilância em Saúde e Ambiente . 2024. “Boletim Epidemiológico.” Brasília, DF. https://www.gov.br/saude/pt‐br/centrais‐de‐conteudo/publicacoes/boletins/epidemiologicos/edicoes/2024/boletim‐epidemiologico‐volume‐55‐no‐07.pdf.

[ajhb70231-bib-0040] Nogueira, B. S. , M. G. S. Guimarães , C. B. e. Braga , et al. 2021. “Fatores associados ao consumo alimentar excessivo de frituras em município da Amazônia ocidental brasileira.” Scientia Naturalis 3, no. 5: 1985–2003.

[ajhb70231-bib-0041] Oliveita, A. M. A. , E. M. M. Cerqueira , J. da Silva Souza , and A. C. de Oliveira . 2003. “Sobrepeso e obesidade infantil: influência de fatores biológicos e ambientais em Feira de Santana, BA.” Arquivos Brasileiros de Endocrinologia e Metabologia 47: 144–150.

[ajhb70231-bib-0042] Palacios, C. , M. Magnus , A. Arrieta , H. Gallardo , R. Tapia , and C. Espinal . 2021. “Obesity in Latin America, a Scoping Review of Public Health Prevention Strategies and an Overview of Their Impact on Obesity Prevention.” Public Health Nutrition 24, no. 15: 5142–5155. 33843569 10.1017/S1368980021001403 PMC11082825

[ajhb70231-bib-0043] Puglisi, M. J. , and M. L. Fernandez . 2022. “The Health Benefits of Egg Protein.” Nutrients 14, no. 14: 2904. 35889862 10.3390/nu14142904 PMC9316657

[ajhb70231-bib-0044] Santana, N. M. T. 2017. “Consumo de álcool e pressão arterial: resultados da linha de base do ELSA‐Brasil.” 2017. Dissertação (Mestrado em Saúde Coletiva)—Centro de Ciências da Saúde, Universidade Federal do Espírito Santo, Vitória ‐ES, 2017.

[ajhb70231-bib-0045] Sellen, D. 1998. “Physical Status: The Use and Interpretation of Anthropometry. Report of a WHO Expert Committee. WHO Technical Report Series No. 854. Pp. 452(WHO, Geneva, 1995.) Swiss Fr 71.00.” Journal of Biosocial Science 30, no. 1: 135–144. 8594834

[ajhb70231-bib-0046] Silva, T. R. , C. Feldmam , M. H. A. Lima , M. R. C. Nobre , and R. Z. L. Domingues . 2006. “Controle de diabetes Mellitus e hipertensão arterial com grupos de intervenção educacional e terapêutica em seguimento ambulatorial de uma Unidade Básica de Saúde.” Saúde e Sociedade 15, no. 3: 180–189.

[ajhb70231-bib-0047] Simões, B. d. S. , G. L. L. Machado‐Coelho , J. L. Pena , and S. N. de Freitas . 2013. “Perfil nutricional dos indígenas Xukuru‐Kariri, Minas Gerais, de acordo com diferentes indicadores antropométricos e de composição corporal.” Ciência & Saúde Coletiva 18: 405–411. 23358766 10.1590/s1413-81232013000200012

[ajhb70231-bib-0048] Sousa, A. P. d. M. , I. C. Pereira , L. d. L. Araujo , M. R. da Rocha , H. M. M. Bandeira , and L. H. d. O. Lima . 2021. “Prevalência e fatores associados ao excesso de peso em adultos nas capitais e no Distrito Federal, Brasil, 2019.” Epidemiologia e Serviços de Saúde 30, no. 3: e2020838. 34287557 10.1590/S1679-49742021000300014

[ajhb70231-bib-0049] Souza Filho, P. W. M. , and M. El‐Robrini . 1996. “Morfologia, processos de sedimentação e litofácies dos ambientes morfo‐sedimentares da planície costeira bragantina, nordeste do Pará, Brasil.” Geonomos 4, no. 2: 1–16.

[ajhb70231-bib-0050] Thomas, M. S. , M. Puglisi , O. Malysheva , et al. 2022. “Eggs Improve Plasma Biomarkers in Patients With Metabolic Syndrome Following a Plant‐Based Diet—A Randomized Crossover Study.” Nutrients 14, no. 10: 2138. 35631279 10.3390/nu14102138 PMC9147178

[ajhb70231-bib-0051] Vander Wal, J. S. , J. M. Marth , P. Khosla , K.‐L. C. Jen , and N. V. Dhurandhar . 2005. “Short‐Term Effect of Eggs on Satiety in Overweight and Obese Subjects.” Journal of the American College of Nutrition 24, no. 6: 510–515. 16373948 10.1080/07315724.2005.10719497

[ajhb70231-bib-0052] WHO . 2003. “Diet, Nutrition and the Prevention of Chronic Diseases: Report of a Joint WHO/FAO Expert Consultation.” WHO Technical Report Series 916: 1–149. 12768890

[ajhb70231-bib-0053] WHO Consultation on Obesity . 2000. “Obesity: Preventing and Managing the Global Epidemic.” World Health Organization Technical Report Series 894: 1–253. 11234459

[ajhb70231-bib-0054] WHO Expert Committee on Physical Status . 1995. “The Use and Interpretation of Anthropometry.” WHO Technical Report Series 854, no. 9: 1–452. 8594834

[ajhb70231-bib-0055] World Health Organization . 2010. “Global Recommendations on Physical Activity for Health.” 60. https://iris.who.int/bitstream/handle/10665/44436/9789242599978_fre.pdf?ua=1. 26180873

[ajhb70231-bib-0056] World Health Organization . 2025. “Obesity and Overweight.” https://www.who.int/news‐room/fact‐sheets/detail/obesity‐and‐overweight.

