# Supplementary material for: Excess Weight Among Adults Living in the Coastal Brazilian Amazon: Prevalence, Determinants, and Interventions
Source: Am J Hum Biol. 2026 Feb 25;38(3):e70231. doi: 10.1002/ajhb.70231 (PMC12935521; doi:10.1002/ajhb.70231)
Supplement: Supplementary file 1 — Table S1: Prevalence and crude prevalence ratio (CPR) of excess weight in adults residing in the Brazilian municipality of Bragança, according to socioeconomic and demographic variables, housing and basic sanitation, dietary habits and behavioral patterns. [file AJHB-38-e70231-s001.docx]

**SUPPLEMENTARY MATERIAL**

Table S1: Prevalence and crude prevalence ratio (CPR) of excess weight in adults residing in the Brazilian municipality of Bragança, according to socioeconomic and demographic variables, housing and basic sanitation, dietary habits and behavioral patterns.

| **Variables** | **Sample** | | | **Excess weight** | | | | **CPR** | | **P** | | |
| --- | --- | --- | --- | --- | --- | --- | --- | --- | --- | --- | --- | --- |
|  | **N** | | **%** | **N** | | **%** | | **(CI 95%)** | |  |  |  |
| **Socioeconomic and demographic** | | | | | | | | | | | | |
| Sex |  | | | | | | | | | | | |
| Female | 175 | | 42.9 | 108 | | 36.9 | | 1.0 | | 0.71 | | |
| Male | 232 | | 57.1 | 150 | | 26.5 | | 27.55 (25.69-27.84) | |  |  |  |
| Age group (years) |  | | | | | | | | | | | |
| 30 – 39 | 125 | | 30.7 | 84 | | 20.6 | | 1.0 | | 0.77 | | |
| 40 – 49 | 123 | | 30.2 | 74 | | 18.2 | | 19.09 (17.55-19.42) | |  |  |  |
| 50 – 59 | 94 | | 23.1 | 63 | | 15.5 | | 16.49 (14.78-16.88) | |  |  |  |
| 60 or more | 65 | | 15.9 | 37 | | 9.1 | | 9.95 (8.3-10.35) | |  |  |  |
| Marital status |  | | | | | | | | | | | |
| Married + Stable Union | 310 | | 76.2 | 200 | | 49.1 | | 1.0 | | 0.61 | | |
| Single + Divorced + Widowed | 97 | | 23.8 | 58 | | 14.3 | | 15.22 (13.61-15.54) | |  |  |  |
| Education |  | | | | | | | | | | | |
| Complete elementary | 122 | | 29.9 | 85 | | 20.8 | | 1.0 | | 0.56 | | |
| Illiterate | 118 | | 28.9 | 73 | | 17.9 | | 18.78 (17.25-19.11) | |  | | |
| High school | 145 | | 35.6 | 90 | | 22.1 | | 22.99 (21.44-24.09) | |  |  |  |
| Higher education | 22 | | 5.4 | 10 | | 2.5 | | 3.15 (2.17-3.75) | |  |  |  |
| Raçe/Ethnicity |  | | | | | | | | | | | |
| Yellow | 1 | | 0.25 | 1 | | 0.25 | | 1.0 | | 0.91 | | |
| Indigenous | 3 | | 0.74 | 2 | | 0.49 | | 1.16 (0.34-3.36) | |  |  |  |
| Mixed | 339 | | 83.3 | 212 | | 52.1 | | 52.73 (51.91-54.17) | |  |  |  |
| White | 52 | | 12.8 | 35 | | 8.6 | | 9.27 (8.4-10.85) | |  |  |  |
| Black | 12 | | 2.95 | 8 | | 1.9 | | 2.57 (1.71-4.26) | |  |  |  |
| Monthly income per minimum wage |  | | | | | | | | | | | |
| Up to 1 | 240 | | 58.9 | 152 | | 37.4 | | 1.0 | | 0.26 | | |
| More than 1 up to 2 | 107 | | 26.3 | 64 | | 15.7 | | 16.64 (14.99-16.96) | |  |  |  |
| More than 2 up to 3 | 31 | | 7.6 | 19 | | 4.7 | | 5.67 (4.1-6.26) | |  |  |  |
| More than 3 up to 4 | 14 | | 3.4 | 13 | | 3.2 | | 4.67 (2.37-5.78) | |  |  |  |
| More than 4 | 15 | | 3.7 | 10 | | 2.5 | | 3.55 (1.94-4.49) | |  |  |  |
| Housing and basic sanitation | | | | | | | | | | | | |
| Walls of the residence | |  | | | | | | | | | | |
| Others | | 41 | 10.1 | | 23 | | 5.7 | | 1.0 | | 0.29 | |
| Brick/masonry | | 366 | 89.9 | | 235 | | 57.7 | | 58.84 (56.82-59.18) | |  |  |
| Water supply | |  | | | | | | | | | | |
| Others | | 301 | 76.9 | | 192 | | 47.2 | | 1.0 | | 0.77 | |
| General network | | 106 | 26.4 | | 66 | | 16.2 | | 17.17 (15.38-17.35) | |  |  |
| Destination of waste | |  | | | | | | | | | | |
| Public collection | | 276 | 67.8 | | 178 | | 43.7 | | 1.0 | | 0.50 | |
| Others | | 131 | 32.2 | | 80 | | 19.7 | | 20.65 (18.89-20.81) | |  |  |
| Destination of household sewage | | | | | | | | | | | | |
| Others | | 400 | 98.3 | | 251 | | 61.7 | | 1.0 | | 0.25 | |
| General network | | 7 | 1.7 | | 7 | | 1.7 | | 3.29 (0.95-4.21) | |  |  |
| **Dietary habits** | | | | | | | | | | | | |
| Eat white rice | |  | | | | | | | | | | |
| 1-2 times | | 13 | 3.2 | | 7 | | 1.7 | 1.0 | | | | 0.59 |
| 3-4 times | | 17 | 4.2 | | 13 | | 3.2 | 4.62 (2.39-5.68) | | | |  |
| 5-7 times | | 361 | 88.7 | | 227 | | 55.8 | 56.96 (55.06-57.64) | | | |  |
| Never | | 16 | 3.9 | | 11 | | 2.7 | 3.97 (1.98-4.97) | | | |  |
| Eat beans | |  | | | | | | | | | | |
| 1-2 times | | 93 | 22.9 | | 63 | | 15.5 | 1.0 | | | | 0.19 |
| 3-4 times | | 87 | 21.4 | | 59 | | 14.5 | 15.51 (13.69-15.74) | | | |  |
| 5-7 times | | 142 | 34.9 | | 80 | | 19.7 | 20.53 (19.2-20.72) | | | |  |
| Never | | 85 | 20.9 | | 56 | | 13.8 | 14.77 (13.02-15.01) | | | |  |
| Eat pasta | |  | | | | | | | | | | |
| 1-2 times | | 175 | 43.0 | | 117 | | 28.8 | 1.0 | | | | 0.45 |
| 3-4 times | | 35 | 8.6 | | 19 | | 4.7 | 5.51 (4.1-5.79) | | | |  |
| 5-7 times | | 24 | 5.9 | | 16 | | 3.9 | 4.89 (3.18-5.27) | | | |  |
| Never | | 173 | 42.5 | | 106 | | 26.1 | 27.02 (25.32-27.18) | | | |  |

| Eat cassava flour |  | | | | | | | |
| --- | --- | --- | --- | --- | --- | --- | --- | --- |
| 1-2 times | 8 | 1.9 | | 5 | 1.2 | | 1.0 | 0.66 |
| 3-4 times | 5 | 1.2 | | 2 | 0.49 | | 1.13 (0.25-2.22) |  |
| 5-7 times | 357 | 87.7 | | 226 | 55.5 | | 56.51 (54.91-57.24) |  |
| Never | 37 | 9.1 | | 25 | 6.1 | | 7.18 (5.5-8.04) |  |
| Eat boiled salad |  | | | | | | | |
| 1-2 times | 151 | 37.1 | | 94 | 23.1 | | 1.0 | 0.83 |
| 3-4 times | 70 | 17.2 | | 46 | 11.3 | | 12.35 (10.45-12.61) |  |
| 5-7 times | 53 | 13.1 | | 36 | 8.9 | | 9.99 (8.04-10.28) |  |
| Never | 133 | 32.7 | | 82 | 20.2 | | 21.19 (19.37-21.39) |  |
| Eat fried egg |  | | | | | | | |
| 1-2 times | 102 | 25.1 | | 56 | 13.8 | | 1.0 | 0.66 |
| 3-4 times | 37 | 9.1 | | 25 | 6.1 | | 7.33 (5.33-8.07) |  |
| 5-7 times | 18 | 4.4 | | 12 | 2.9 | | 4.11 (2.25-5.17) |  |
| Never | 250 | 61.4 | | 165 | 40.5 | | 41.07 (39.61-42.13) |  |
| Eat tubers |  | | | | | | | |
| 1-2 times | 40 | 9.8 | | 22 | 5.4 | | 1.0 | 0.67 |
| 3-4 times | 27 | 6.6 | | 17 | 4.2 | | 5.34 (3.42-6.06) |  |
| 5-7 times | 13 | 3.2 | | 9 | 2.2 | | 3.46 (1.41-4.22) |  |
| Never | 327 | 80.3 | | 210 | 51.6 | | 52.77 (50.71-53.12) |  |
| Eat fruits |  | | | | | | | |
| 1-2 times | 32 | 7.9 | | 16 | 3.9 | | 1.0 | 0.35 |
| 3-4 times | 83 | 20.4 | | 55 | 13.5 | | 14.83 (12.56-15.36) |  |
| 5-7 times | 222 | 54.6 | | 144 | 35.4 | | 36.62 (34.45-37.18) |  |
| Never | 70 | 17.2 | | 43 | 10.6 | | 11.82 (9.73-12.34) |  |
| Eat bread |  | | | | | | | |
| 1-2 times | 33 | 8.11 | | 25 | 6.1 | | 1.0 | 0.59 |
| 3-4 times | 29 | 7.13 | | 17 | 4.2 | | 4.97 (3.67-5.33) |  |
| 5-7 times | 289 | 71.0 | | 186 | 44.8 | | 45.63 (44.16-45.88) |  |
| Never | 56 | 13.8 | | 33 | 8.1 | | 8.88 (7.53-9.17) |  |
| Drink whole milk |  | | | | | | | |
| 1-2 times | 20 | 4.9 | | 16 | 3.9 | | 1.0 | 0.44 |
| 3-4 times | 19 | 4.7 | | 12 | 2.9 | | 3.69 (2.04-4.14) |  |
| 5-7 times | 284 | 69.8 | | 180 | 44.2 | | 44.99 (43.62-45.28) |  |
| Never | 84 | 20.6 | | 50 | 12.3 | | 13.04 (11.78-13.34) |  |
| Eat fresh beef |  | | | | | | |  |
| 1-2 times | 136 | | 33.4 | 83 | | 20.4 | 1.0 |  |
| 3-4 times | 186 | | 45.7 | 116 | | 28.5 | 29.52(27.64-29.71) |  |
| 5-7 times | 50 | | 12.3 | 36 | | 8.9 | 10.08 (7.97-10.39) |  |
| Never | 35 | | 8.6 | 23 | | 5.7 | 6.77 (4.89-7.13) |  |
| Eat chicken |  | | | | | | | |
| 1-2 times | 208 | | 51.1 | 131 | | 32.2 | 1.0 | 0.56 |
| 3-4 times | 117 | | 28.8 | 78 | | 19.2 | 20.25 (18.31-20.45) |  |
| 5-7 times | 15 | | 3.69 | 7 | | 1.72 | 2.46 (1.26-2.89) |  |
| Never | 67 | | 16.5 | 42 | | 10.3 | 11.29 (9.25-11.52) |  |
| Eat fresh fish |  | | | | | | | |
| 1-2 times | 127 | | 31.2 | 76 | | 18.7 | 1.0 | 0.40 |
| 3-4 times | 159 | | 39.1 | 108 | | 26.5 | 27.63 (25.55-27.86) |  |
| 5-7 times | 80 | | 19.7 | 47 | | 11.6 | 12.58 (10.82-12.82) |  |
| Never | 41 | | 10.1 | 27 | | 6.6 | 7.07 (5.76-8.03) |  |
| Eat salted fish |  | | | | | | | |
| 1-2 times | 43 | | 10.6 | 23 | | 5.7 | 1.0 | 0.12 |
| 3-4 times | 16 | | 3.9 | 9 | | 2.2 | 3.25 (1.53-3.88) |  |
| 5-7 times | 10 | | 2.5 | 4 | | 0.9 | 1.65 (0.51-2.32) |  |
| Never | 338 | | 83.1 | 222 | | 54.6 | 55.83 (53.65-56.19) |  |
| Eat seafood |  | | | | | | | |
| 1-2 times | 76 | 18.7 | | 43 | 10.6 | | 1.0 | 0.18 |
| 3-4 times | 11 | 2.7 | | 10 | 2.5 | | 4.11 (1.44-4.94) |  |
| 5-7 times | 4 | 0.9 | | 3 | 0.7 | | 2.02 (0.05-3.04) |  |
| Never | 316 | 77.4 | | 202 | 49.6 | | 50.73 (48.68-50.98) |  |
| Drink açaí |  | | | | | | | |
| 1-2 times | 101 | 24.8 | | 68 | 16.7 | | 1.0 | 0.53 |
| 3-4 times | 27 | 6.6 | | 19 | 4.7 | | 5.76 (3.93-6.12) |  |
| 5-7 times | 15 | 3.7 | | 8 | 1.9 | | 2.69 (1.39-3.14) |  |
| Never | 264 | 64.9 | | 163 | 40.1 | | 41.02 (39.33-41.18) |  |
| Drink soda |  | | | | | | | |
| 1-2 times | 76 | 18.7 | | 48 | 11.8 | | 1.0 | 0.86 |
| 3-4 times | 30 | 7.4 | | 19 | 4.7 | | 5.71 (3.97-6.09) |  |
| 5-7 times | 9 | 2.2 | | 7 | 1.7 | | 2.93 (0.94-3.69) |  |
| Never | 292 | 71.4 | | 184 | 45.2 | | 46.19 (44.38-46.41) |  |
| Drink artificial juice |  | | | | | | | |
| 1-2 times | 34 | 8.4 | | 20 | 4.9 | | 1.0 | 0.70 |
| 3-4 times | 24 | 5.9 | | 13 | 3.2 | | 4.12 (2.06-4.61) |  |
| 5-7 times | 26 | 6.4 | | 17 | 4.2 | | 5.31 (3.45-5.85) |  |
| Never | 323 | 79.4 | | 208 | 51.1 | | 52.19 (50.27-52.55) |  |
| Eat cakes and/or pies |  | | | | | | | |
| 1-2 times | 102 | 25.1 | | 69 | 16.9 | | 1.0 | 0.71 |
| 3-4 times | 7 | 1.7 | | 4 | 0.9 | | 1.74 (0.44-2.46) |  |
| 5-7 times | 4 | 0.9 | | 3 | 0.7 | | 1.81 (0.15-2.94) |  |
| Never | 294 | 72.2 | | 182 | 44.7 | | 45.62 (43.93-45.78) |  |
| Eat fried snacks |  | | | | | | | |
| 1-2 times | 58 | 14.3 | | 41 | 10.1 | | 1.0 | 0.47 |
| 3-4 times | 9 | 2.2 | | 7 | 1.7 | | 2.08 (1.02-3.49) |  |
| 5-7 times | 12 | 2.9 | | 8 | 1.9 | | 2.84 (1.31-3.39) |  |
| Never | 328 | 80.6 | | 202 | 49.6 | | 50.47 (48.89-50.67) |  |
| Eat processed meats |  | | | | | | | |
| 1-2 times | 71 | 17.4 | | 44 | 10.8 | | 1.0 | 0.21 |
| 3-4 times | 7 | 1.7 | | 7 | 1.7 | | 3.31 (0.71-4.32) |  |
| 5-7 times | 2 | 0.5 | | 2 | 0.5 | | 2.11 (0.18-4.31) |  |
| Never | 327 | 80.3 | | 205 | 50.4 | | 51.41 (49.58-51.63) |  |
| Use sweetener |  | | | | | | | |
| No | 362 | 88.9 | | 227 | 55.8 | | 1.0 | 0.42 |
| Yes | 45 | 11.1 | | 31 | 7.6 | | 8.69 (6.73-8.98) |  |
| Use industrial seasoning |  | | | | | | | |
| No | 247 | 60.7 | | 163 | 40.1 | | 1.0 | 0.18 |
| Yes | 160 | 39.3 | | 95 | 23.3 | | 24.19 (22.53-24.35) |  |
| **Behavioral patterns** | | | | | | | | |
| Leisure walk | | | | | | | | |
| No | 368 | 90.4 | | 230 | 56.5 | | 1.0 | 0.26 |
| Yes | 39 | 9.6 | | 28 | 6.9 | | 8.04 (5.99-8.36) |  |

| Biking to work | | | | | | |
| --- | --- | --- | --- | --- | --- | --- |
| No | 301 | 73.9 | 193 | 47.4 | 1.0 | 0.61 |
| Yes | 106 | 26.1 | 65 | 15.9 | 16.86 (15.09-17.03) |  |
| Leisure cycling |  | | | | | |
| No | 399 | 98.0 | 254 | 62.4 | 1.0 | 0.41 |
| Yes | 8 | 1.9 | 4 | 0.9 | 1.68 (0.47-2.33) |  |
| Practices sports |  | | | | | |
| No | 349 | 85.8 | 224 | 55.0 | 1.0 | 0.41 |
| Yes | 58 | 14.3 | 34 | 8.4 | 9.31 (7.67-9.53) |  |
| Currently consumes alcoholic beverages | | | | | | |
| No | 257 | 63.1 | 160 | 39.3 | 1.0 | 0.53 |
| Yes | 150 | 36.9 | 98 | 24.1 | 25.15 (23.02-25.32) |  |
| Currently use tobacco | | | | | | |
| No | 351 | 86.2 | 230 | 56.5 | 1.0 | 0.16 |
| Yes | 56 | 13.8 | 28 | 6.9 | 7.66 (6.39-8.03) |  |
